# Data on chemical characteristics of waters in two boreal *Sphagnum* mires (North-Western Russia)

**DOI:** 10.1016/j.dib.2019.104928

**Published:** 2019-12-04

**Authors:** Dmitriy A. Philippov, Victoria V. Yurchenko

**Affiliations:** Papanin Institute for Biology of Inland Waters Russian Academy of Sciences, Russia

**Keywords:** Natural waters, Spectrophotometry, Solids concentration, Mire water bodies, Raised bog, Wetland

## Abstract

The dataset contains chemical parameters of waters in different mire water bodies (fen strip, bog stream, *Sphagnum* hollows, hollow-pools, intra-mire lakes, drainage way). Data were collected once a month from May till September 2012 and in May, July and September 2013 and 2014 in Shichengskoe and Alekseevskoe-1 mires (Vologda Region, Russia). Water samples were kept in a cooling bag and transported to the laboratory within a day. Prior to analyses, water samples were filtered (pore size 90 μm). Colour of water, pH, permanganate value, dry residues, and total iron, manganese, carbonate, phosphate, sulphate and nitrate ion concentrations were measured. Data were obtained by the atomic absorption spectrometry and spectrophotometric and titrimetric methods. The pH values varied from 3.7 in *Sphagnum* hollows to 6.9 in a bog stream and 7.2 in a primary intra-mire lake. The minimum permanganate value of 5.6 mg O/L was registered in a bog stream, the maximum of 150.4 mg O/L in a weakly waterlogged *Sphagnum* hollow. Dry residue values varied in a range of 35 mg/L in a large hollow-pool to 315 mg/L in a flow-through fen strip. The data are useful for investigating chemical composition of waters in different mire water bodies and the heterogeneity of these abiotic factors.

**Table:** Specifications Table

Subject	Environmental Chemistry
Specific subject area	Hydrochemistry
Type of data	Tables
How data were acquired	UNICO-1201 spectrophotometer (INICO, USA) was used to measure colour of water and phosphate, sulphate and nitrate ion concentrations. Sartorius Basic Meter PB-11 (Sartorius, USA) was used to determine pH values. Atomic absorption Spektr-5 spectrometer (Soyuztsvetmetavtomatika JSC, Russia) was used to analyze total iron and manganese concentrations.
Data format	Raw
Parameters for data collection	Water samples were stored at cool temperature and transported to the laboratory within a day. Prior to analyses water samples were filtered through a 90 μm filter. Atomic absorption spectrometry and spectrophotometric and titrimetric methods were used to obtain the data.
Description of data collection	Data were collected once a month from May till September 2012 and in May, July and September 2013 and 2014. Water level and surface temperature were determined in situ. Colour of water, pH, permanganate value, dry residues, concentrations of total iron, manganese, and carbonate, phosphate, sulphate and nitrate ions in water were measured in laboratory.
Data source location	Mires Shichengskoe and Alekseevskoe-1 in the Vologda Region, Russia
Data accessibility	With the article
Related research article	D.A. Philippov, Hydrochemical characteristics of mire water tracks (by the example of Shichengskoe raised bog, Vologda Region), Water: Chemistry and Ecology. 7 (2014) 10–17 (in Russian) [1].

**Table:** 

**Value of the Data** • These data are useful for investigating abiotic factors in mire water bodies and, especially their spatial and temporal variability. • The data are of particular value to the mire ecologists since the data show differences in hydrochemical parameters within mire ecosystems. • The dataset can be useful to the environmental chemists as it provides new cases for meta-analysis of chemical composition of natural waters. • The dataset can be useful for further insights on abiotic heterogeneity in mire ecosystems as well as investigation of the associations between aquatic biodiversity and chemical composition of natural waters in wetlands. • The data are beneficial for further efforts to create a mathematical model of a mire ecosystem. • The data can be used in assessment of water quality in mire water bodies and their suitability for water supply.

## 1 Data

**Fig. 1:**
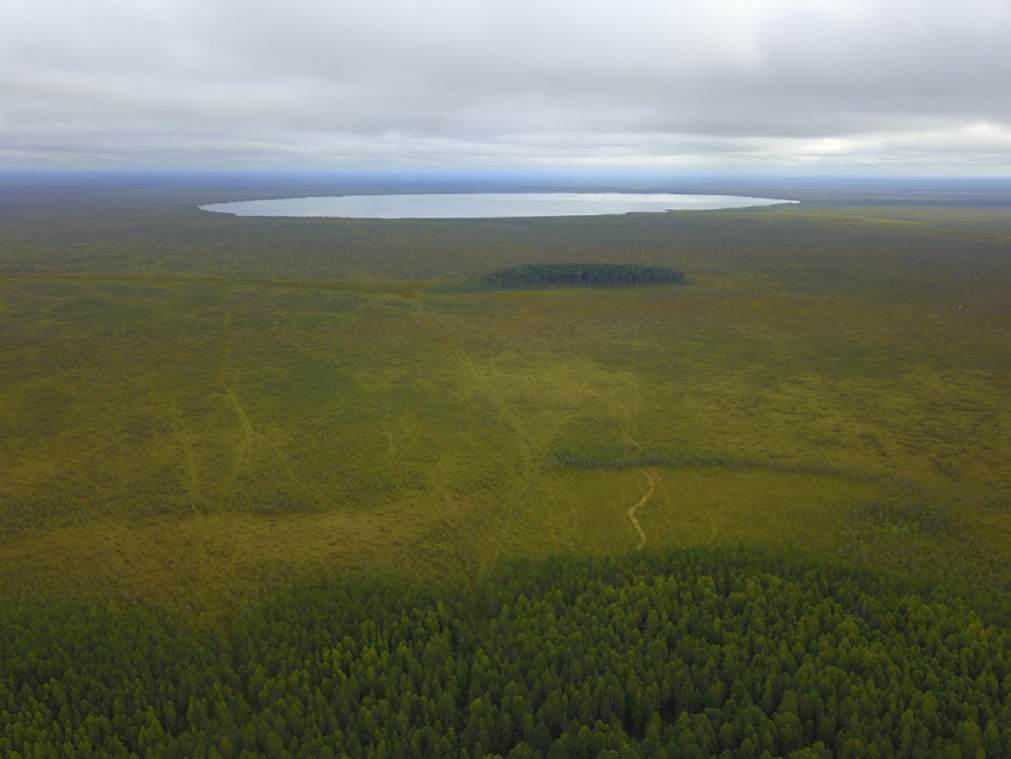
Data collection area. General view of Shichengskoe mire and intra-mire lake Shichengskoe. Aerial surveys performed by D.A. Philippov using a DJI Mavic Pro quadcopter on 19 August 2019.

**Fig. 2:**
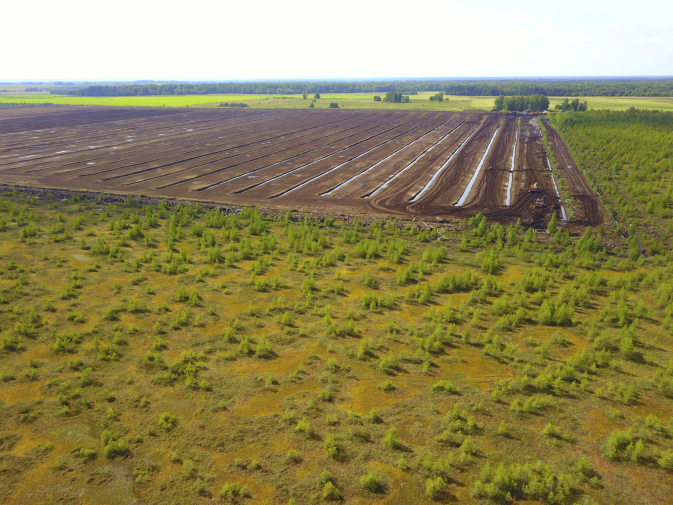
Data collection area. General view of Alekseevskoe-1 mire: natural (undisturbed) part of the mire in the foreground, a peat-cutting site in the background. Aerial surveys performed by D.A. Philippov using a DJI Mavic Pro quadcopter on 20 August 2019.

**Fig. 3:**
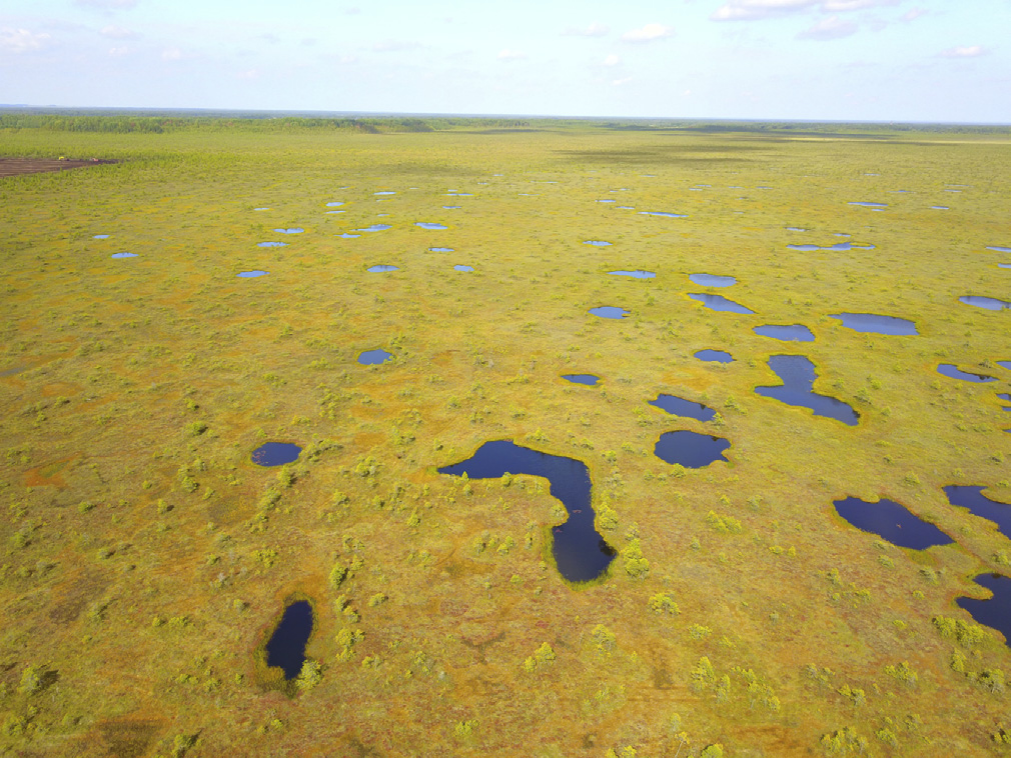
Data collection area. General view of ridge-hollow and ridge-hollow-pool complexes in Alekseevskoe-1 mire. Aerial surveys performed by D.A. Philippov using a DJI Mavic Pro quadcopter on 20 August 2019.

Table 1 provides general description of the sampling sites located in two wetlands, Shichengskoe and Alekseevskoe-1 mires. Table 2 presents data on colour of water, pH, permanganate value, dry residues, concentrations of total iron, manganese, and carbonate, phosphate, sulphate and nitrate ions in water samples collected in a fen strip, a bog stream, and a Sphagnum hollow in Shichengskoe mire in 2012 and 2013. Table 3 summarizes hydrochemical data for a fen strip, a bog stream, Sphagnum hollows, hollow-pools, intra-mire lakes, and a drainage way in Shichengskoe and Alekseevskoe-1 mires in 2014. Fig. 1, Fig. 2, Fig. 3 show the general view of the mires.

**Table 1:** Sampling sites and dates of sampling.

Data collection area	Mire waterbody	Sampling site ID	Coordinates	Sampling date				
				May	June	July	August	September
Vologda Region, Syamzha District, Shichengskoe Mire	flow-through fen strip	S1	59°56′51″ N 41°17′09″ E	2012-05-27 2013-05-26 2014-05-23	2012-06-27	2012-07-27 2013-07-15 2014-07-18	2012-08-27	2012-09-27 2013-09-18 2014-09-19
	Sphagnum hollow (weakly waterlogged)	S2	59°56′31″ N 41°16′54″ E	2012-05-27 2013-05-26 2014-05-23	2012-06-27	2012-07-27 2013-07-15 2014-07-18	2012-08-27	2012-09-27 2013-09-18 2014-09-19
	bog stream	S3	59°56′26″ N 41°16′05″ E	2012-05-27 2013-05-26 2014-05-23	2012-06-27	2012-07-27 2013-07-15 2014-07-18	2012-08-27	2012-09-27 2013-09-18 2014-09-19
	Lake Shichengskoe (primary intra-mire lake)	S4	59°56′59″ N 41°19′15″ E	–	–	2012-07-28 2014-07-16	–	–
	Lake Polyanok (primary intra-mire lake)	S5	59°55′58″ N 41°31′41″ E	–	–	2014-07-14	–	–
Vologda Region, Sokol District, Alekseevskoe-1 Mire	Sphagnum hollow (weakly waterlogged)	A1	59°27′09″ N 40°30′36″ E	2014-05-25	–	2014-07-20	–	2014-09-21
	Sphagnum hollow (moderately waterlogged)	A2	59°27′11″ N 40°30′46″ E	2014-05-25	–	2014-07-20	–	2014-09-21
	Sphagnum hollow (strongly waterlogged)	A3	59°27′11″ N 40°30′55″ E	2014-05-25	–	2014-07-20	–	2014-09-21
	hollow-pool (small)	A4	59°27′12″ N 40°30′58″ E	2014-05-25	–	2014-07-20	–	2014-09-21
	hollow-pool (medium)	A5	59°27′11″ N 40°30′59″ E	2014-05-25	–	2014-07-20	–	2014-09-21
	hollow-pool (large)	A6	59°27′07″ N 40°31′03″ E	2014-05-25	–	2014-07-20	–	2014-09-21
	drainage way	A7	59°27′10″ N 40°30′32″ E	2014-05-25	–	2014-07-20	–	2014-09-21

**Table 2:** Chemical characteristics of water in different mire water bodies of Shichengskoe mire in 2012 and 2013.

Parameter, units	Sampling date (see Table 1)	2012				2013		
		Sample ID				Sample ID		
		S1	S2	S3	S4	S1	S2	S3
Colour of water, PCU	May	269	89	258	–	162	75	236
	June	210	121	350	–	–	–	–
	July	286	124	432	119	328	118	249
	August	310	127	210	–	–	–	–
	September	173	115	369	–	153	102	137
pH	May	4.9	4.3	6.4	–	5.6	4.6	6.2
	June	5.5	4.1	6.1	–	–	–	–
	July	5.3	4.1	6.9	7.1	5.7	4.4	6.3
	August	5.2	4	6.5	–	–	–	–
	September	5.1	4	5.9	–	5.8	4.9	6.7
Permanganate value, mg O/L	May	45.6	32	43.2	–	27.2	19.6	32
	June	46.4	39.2	5.6	–	–	–	–
	July	64.8	64.8	64.8	64.8	72.8	22.8	49.6
	August	96.8	68.8	50.4	–	–	–	–
	September	45.2	45.6	71.2	–	84	88	50.4
Dry residues, mg/L	May	77	94	49	–	90	85	107
	June	114	104	103	–	–	–	–
	July	171	144	162	128	315	242	244
	August	205	162	237	–	–	–	–
	September	118	86	144	–	184	215	303
Total iron, mg/L	May	5.35	0.12	0.56	–	1.38	0.05	0.41
	June	4.2	0.19	0.92	–	–	–	–
	July	9.7	0.2	1.6	0.3	16.9	0.22	3.99
	August	2.5	0.13	2.4	–	–	–	–
	September	1.4	0.08	0.9	–	2.81	<0.1	6.2
Manganese, mg/L	May	0.21	<0.01	0.02	–	0.32	0.02	0.01
	June	0.34	<0.01	0.02	–	–	–	–
	July	0.49	0.03	0.11	0.04	0.48	0.02	0.75
	August	0.29	0.03	0.49	–	–	–	–
	September	0.24	0.02	0.06	–	0.4	0.03	0.68
Carbonate ions, mg/L	May	9	6	21	–	12	18	21
	June	6	3	45	–	–	–	–
	July	12	6	30	6	78	30	138
	August	18	12	162	–	–	–	–
	September	9	3	9	–	42	24	237
Phosphate ions, mg/L	May	0.14	<0.05	0.28	–	<0.05	<0.05	<0.05
	June	<0.05	0.11	0.16	–	–	–	–
	July	0.39	0.07	0.24	0.18	<0.05	<0.05	0.12
	August	1.51	<0.05	3.25	–	–	–	–
	September	0.23	0.07	0.25	–	<0.05	<0.05	0.58
Nitrate ions, mg/L	May	0.2	0.2	0.3	–	0.5	0.4	0.4
	June	0.9	0.5	0.6	–	–	–	–
	July	0.4	0.3	0.4	0.4	0.4	0.4	0.3
	August	1.1	1.1	0.5	–	–	–	–
	September	0.3	0.3	0.3	–	0.5	0.6	0.3
Temperature, °C	May	13	14	9	–	15	16	11
	June	16	18	17	–	–	–	–
	July	18	22	16	24	19	23	17
	August	13	16	12	–	–	–	–
	September	9	11	9	–	12	13	12
Water level, cm Min Max	May	10 20	0 2	35 125	120 230	15 25	0 5	55 145
	June	5 10	−4 −1	30 120	120 230	–	–	–
	July	1 5	−5 −3	25 115	120 230	−5 −3	−10 −5	20 110
	August	−5 0	−12 −10	20 110	120 230	–	–	–
	September	5 15	−2 1	45 135	120 230	−5 0	−5 −3	25 115

**Table 3:** Chemical characteristics of water in different mire water bodies of Shichengskoe and Alekseevskoe-1 mires in 2014.

Parameter, units	Sampling date (see Table 1)	Sample ID											
		S1	S2	S3	S4	S5	A1	A2	A3	A4	A5	A6	A7
Colour of water, PCU	May	387	111	314	–	–	254	152	140	105	88	44	243
	July	403	139	309	110	63	337	262	183	80	112	47	220
	September	292	90	276	–	–	431	197	248	87	83	91	264
pH	May	5.7	4.3	5.6	–	–	3.8	3.9	3.9	4.1	4.4	4.6	4.7
	July	5.6	4.1	6.5	6.5	7.2	3.9	3.7	4.0	4.2	4.2	4.6	5.2
	September	5.2	3.9	6.8	–	–	3.7	3.8	3.9	4.3	4.3	4.6	5.5
Permanganate value, mg O/L	May	77	47	6	–	–	68	46.4	43.2	31.2	66.4	12.8	60.8
	July	74	89.6	77.6	40	22.4	150.4	118.4	59.2	73.6	30.4	18.4	73.6
	September	80	38	38	–	–	120	60.8	61.6	52.8	28.8	14.1	68
Dry residues, mg/L	May	107	126	122	–	–	122	118	95	86	72	35	138
	July	183	160	250	120	123	255	225	109	103	75	50	146
	September	140	199	204	–	–	283	200	168	169	80	56	147
Total iron, mg/L	May	12.14	0.19	0.52	–	–	0.24	0.26	0.14	0.06	0.07	0.05	0.26
	July	12.97	0.21	5.72	0.3	0.1	0.79	0.52	0.26	0.1	0.1	0.16	0.46
	September	7.73	0.23	1.37	–	–	0.71	0.48	0.18	0.47	0.18	0.17	0.8
Manganese, mg/L	May	0.79	0.02	0.08	–	–	0.01	0.02	<0.01	<0.01	<0.01	0.01	0.01
	July	0.3	0.04	1.94	0.03	<0.01	0.02	0.05	<0.01	0.02	<0.01	0.01	0.02
	September	0.16	0.01	0.06	–	–	<0.01	0.02	0.02	0.06	<0.01	0.01	<0.01
Carbonate ions, mg/L	May	18	<6	30	–	–	<6	<6	<6	<6	<6	<6	<6
	July	78	<6	144	24	60	<6	<6	<6	<6	6	<6	9
	September	78	12	84	–	–	6	<6	6	<6	6	6	18
Phosphate ions, mg/L	May	0.07	0.19	0.11	–	–	0.07	<0.05	0.05	<0.05	<0.05	<0.05	0.09
	July	0.06	0.06	0.45	<0.05	<0.05	0.08	<0.05	<0.05	<0.05	<0.05	<0.05	0.08
	September	0.15	<0.05	0.12	–	–	0.25	<0.05	<0.05	<0.05	<0.05	<0.05	0.11
Sulphate ions, mg/L	May	<10	17.5	<10	–	–	<10	<10	<10	<10	<10	<10	<10
	July	<10	<10	10.5	13.2	<10	11.5	11.7	12	12	23.5	12.2	<10
	September	<10	<10	<10	–	–	<10	<10	<10	<10	<10	<10	<10
Nitrate ions, mg/L	May	0.2	0.3	0.2	–	–	0.3	0.3	0.1	0.3	0.3	0.3	0.2
	July	0.4	0.4	0.3	0.1	2.6	0.7	0.8	0.4	0.4	0.4	0.3	0.5
	September	0.5	0.1	0.5	–	–	0.9	0.8	0.6	0.6	0.9	0.5	0.6
Temperature, °C	May	21	23	15	–	–	16	17	18	20	21	23	17
	July	18	24	17	23	26	14	15	16	18	20	22	20
	September	11	13	10	–	–	6	6.5	7	7	10	13	11
Water level, cm Min Max	May	5 10	−2 0	50 140	120 230	300 700	−10 −5	−5 5	2 5	50 100	100 150	200 250	15 40
	July	−5 1	−5 −3	25 115	120 230	300 700	−15 −12	−5 −4	−1 0	40 90	100 150	200 250	10 35
	September	−8 0	−7 −5	35 125	120 230	300 700	−14 −11	−7 −5	−5 1	20 80	90 140	190 240	15 40

## 2 Experimental design, materials, and methods

Water level (or depth in a stream, hollow-pools and lakes) was measured by a steel ruler or a rope-weight gauge. Five measurements were made per sampling plot; min and max values are given in the article (Table 2, Table 3). Temperature in the water surface layer was measured using a standard mercury filled centigrade thermometer. Water samples were collected in clean plastic bottles, kept in a cooling bag and delivered to the laboratory within a day. Water samples were then filtered through a 0.90 μm filter.

Colour of water was measured by the Platinum–Cobalt method (e.g. Ref. [2]) at 413 nm using a UNICO-1201 spectrophotometer (INICO, USA). The pH was measured using a Sartorius Basic Meter PB-11 (Sartorius, USA). Permanganate value was determined by a modification of the standard procedure [3]. Water samples were incubated with acidified potassium permanganate for 10 minutes at 100 °C. The remaining unreduced permanganate is determined by addition of excess oxalic acid and back titration with potassium permanganate. The content of dry residues in water samples was obtained after evaporation at 100 °C. Analyses of total iron and manganese were carried out by atomic absorption spectrometry using a Spektr-5 spectrometer (Soyuztsvetmetavtomatika JSC, Russia). Carbonate content was measured as carbonate alkalinity by the potentiometric titration up to pH 5.4. Phosphate ion concentrations was determined by the photometric procedure with ammonium orthomolybdate at 690 nm. Sulphate ion concentrations was measured by the turbidimetric procedure at 650 nm. Nitrate ion concentrations was measured by the photometric procedure with salicylic acid at 410 nm. A UNICO-1201 spectrophotometer was used for these analyses.

## References

[1] Philippov D.A. (2014). Hydrochemical characteristics of mire water tracks (by the example of Shichengskoe raised bog, Vologda Region). Water: Chem. Ecol..

[2] Thermo Scientific (2013). Application Note, Meter Log #133. https://assets.thermofisher.com/TFS-Assets/LSG/Application-Notes/Log-133-Color-in-Water-and-Wastewater-Platinum-Cobalt-at-455-nm.pdf.

[3] Methods for the examination of waters and associated materials (1983). The Permanganate Index and Permanganate Value Tests for Waters and Effluents.

